# Supplementary material for: Social stratification without genetic differentiation at the site of Kulubnarti in Christian Period Nubia
Source: Nat Commun. 2021 Dec 14;12:7283. doi: 10.1038/s41467-021-27356-8 (PMC8671435; doi:10.1038/s41467-021-27356-8)
Supplement: Supplementary file 3 — Description of Additional Supplementary Files [file 41467_2021_27356_MOESM3_ESM.pdf]

Description of Additional Supplementary Files

File Name: Supplementary Data 1
Description: **Skeletal material screened for analysis in the present study.** Screening at UCD took place via shotgun sequencing on a MiSeq or NextSeq500 instrument; individuals screened at UCD were not given a Master ID (see Supplementary Note 2 for additional information). Individuals labeled with asterisk (*) are lower-coverage first-degree relatives of another individual in our dataset and were not included in statistical analyses.

File Name: Supplementary Data 2
Description: **Library-level information for the newly reported individuals in this study.**

File Name: Supplementary Data 3
Description: **Ancient West Eurasian and African individuals used in the reference dataset of this work.**

File Name: Supplementary Data 4
Description: **Individuals included in PCA.**

File Name: Supplementary Data 5
Description: **Admixture f3-statistics show a history of admixture between Nilotic-related ancestry (A) and West Eurasian-related ancestry (B) at Kulubnarti.**

File Name: Supplementary Data 6
Description: **Individual f4-statistics testing for excess Nilotic- or West Eurasian-related ancestry.** Results with $|Z| > 5.0$ in bold (interpreted as genetic oultlier), results with $|Z| > 3.0$ in italics. Individuals labeled with asterisk (*) are lower-coverage first-degree relatives of another individual in our dataset.

File Name: Supplementary Data 7
Description: **Results of qpAdm modeling; models interpreted as significant (p>0.05) in bold.** O9 reference set: Mbuti, Onge, Chukchi, Karitiana, Papuan, Han, Ust Ishim, MA1, Kostenki14

File Name: Supplementary Data 8
Description: **qpAdm estimates of individual ancestry proportions. Individuals in bold are considered genetic outliers in this work.** Reference set: O9 (Mbuti, Onge, Chukchi, Karitiana, Papuan, Han, Ust Ishim, MA1, Kostenki14) + Anatolia_EBA

File Name: Supplementary Data 9
Description: **qpWave modelling; models interpreted as significant (p>0.05) in bold.**

File Name: Supplementary Data 10
Description: **Group f4-statistics testing for excess Nilotic- or West Eurasian-related ancestry in the S and R cemeteries.**

File Name: Supplementary Data 11
Description: **Estimated dates of admixture at Kulubnarti.** Only estimates with |Z|>2.8 reported (corresponding to a 99.5% CI).

File Name: Supplementary Data 12
Description: **mtDNA haplogroup calls and mutations. Calls made with Haplogrep Classify v2.2.8.**

File Name: Supplementary Data 13
Description: **Y chromosome haplogroups.**

File Name: Supplementary Data 14
Description: **Estimated dates of admixture for three present-day Nubian groups.** Models in bold discussed in main text. Models using Nuer and TSI as sources included here for comparability with Hollfelder et al. (2017).
