# Supplementary material for: Social stratification without genetic differentiation at the site of Kulubnarti in Christian Period Nubia
Source: Nat Commun. 2021 Dec 14;12:7283. doi: 10.1038/s41467-021-27356-8 (PMC8671435; doi:10.1038/s41467-021-27356-8)
Supplement: Supplementary file 18 — Reporting Summary [file 41467_2021_27356_MOESM18_ESM.pdf]

nature research

Corresponding author(s): Kendra Sirak

Last updated by author(s): Oct 26, 2021

Reporting Summary

Nature Research wishes to improve the reproducibility of the work that we publish. This form provides structure for consistency and transparency in reporting. For further information on Nature Research policies, see our Editorial Policies and the Editorial Policy Checklist.

Statistics

For all statistical analyses, confirm that the following items are present in the figure legend, table legend, main text, or Methods section.

n/a	Confirmed	
☐	☒	The exact sample size (*n*) for each experimental group/condition, given as a discrete number and unit of measurement
☐	☒	A statement on whether measurements were taken from distinct samples or whether the same sample was measured repeatedly
☐	☒	The statistical test(s) used AND whether they are one- or two-sided<br>*Only common tests should be described solely by name; describe more complex techniques in the Methods section.*
☒	☐	A description of all covariates tested
☐	☒	A description of any assumptions or corrections, such as tests of normality and adjustment for multiple comparisons
☐	☒	A full description of the statistical parameters including central tendency (e.g. means) or other basic estimates (e.g. regression coefficient) AND variation (e.g. standard deviation) or associated estimates of uncertainty (e.g. confidence intervals)
☐	☒	For null hypothesis testing, the test statistic (e.g. *F*, *t*, *r*) with confidence intervals, effect sizes, degrees of freedom and *P* value noted<br>*Give P values as exact values whenever suitable.*
☒	☐	For Bayesian analysis, information on the choice of priors and Markov chain Monte Carlo settings
☒	☐	For hierarchical and complex designs, identification of the appropriate level for tests and full reporting of outcomes
☒	☐	Estimates of effect sizes (e.g. Cohen's *d*, Pearson's *r*), indicating how they were calculated

Our web collection on statistics for biologists contains articles on many of the points above.

Software and code

Policy information about availability of computer code

Data collection	BWA v.0.7.15-r1140; Picard MarkDuplicates v2.17.10; contamMix v.1.0-12; OxCal v4.4
Data analysis	samtools v.1.3.1; bcftools v.1.10.2; Haplogrep Classify v.2.2.8; hapROH; smartpca v.181602; ADMIXTOOLS v6.0; DATES v.3520; Yfull version 8.09; ISOGG version 15.73; PhyloTree version 17; https://github.com/DReichLab/ADNA-Tools

For manuscripts utilizing custom algorithms or software that are central to the research but not yet described in published literature, software must be made available to editors and reviewers. We strongly encourage code deposition in a community repository (e.g. GitHub). See the Nature Research guidelines for submitting code & software for further information.

Data

Policy information about availability of data

All manuscripts must include a data availability statement. This statement should provide the following information, where applicable:

- Accession codes, unique identifiers, or web links for publicly available datasets
- A list of figures that have associated raw data
- A description of any restrictions on data availability

Aligned sequences are available through the European Nucleotide Archive under accession number PRJEB42975 (https://www.ebi.ac.uk/ena/browser/view/PRJEB42975). Genotype data used in analysis is also available at https://reich.hms.harvard.edu/datasets. Previously published ancient data used in this study are available under accession numbers PRJEB37057, PRJEB24794, PRJEB8448, PRJEB11450, PRJEB22652, PRJEB22629, PRJEB32466, PRJEB27215, PRJEB6272, PRJEB14455, PRJEB20914, PRJEB30874, and ERP017224 and at https://reich.hms.harvard.edu/datasets. Genotype data from present-day individuals, publicly available, were accessed as indicated in their corresponding original publications (Fan et al. 2019, Hollfelder et al. 2017, Mallick et al. 2016, Meyer et al. 2012, Pagani et al. 2012, Prufer et al. 2013, Skoglund et al. 2015, The 1000 Genomes Project Consortium 2015.) The hg19 reference genome is publicly available under GenBank assembly accession GCA_000001405.1.

Field-specific reporting

Please select the one below that is the best fit for your research. If you are not sure, read the appropriate sections before making your selection.

☐ Life sciences ☐ Behavioural & social sciences ☒ Ecological, evolutionary & environmental sciences

For a reference copy of the document with all sections, see nature.com/documents/nr-reporting-summary-flat.pdf

Ecological, evolutionary & environmental sciences study design

All studies must disclose on these points even when the disclosure is negative.

Study description	Genetic analyses were performed on DNA data generated from ancient human skeletons; genetic data from 66 new ancient individuals is reported. Population genetic statistics, primarily testing historical relationships by measuring allele-sharing patterns across populations, were computed using genome-wide SNP genotypes. The dataset was analyzed to investigate the genetic makeup of a Christian Period Sudanese Nubian group.
Research sample	Genome-wide SNP data was obtained from 66 individuals from the Sudanese Nubian Christian Period site of Kulubnarti. DNA was extracted and sequenced in dedicated ancient DNA facilities. Both male (n=30) and female (n=36) individuals from Kulubnarti were analyzed; the genetic sex of each individual is provided in Supplementary Data 1. The analyzed individuals ranged from 7 months in utero to ~27 years old at time of death; age at death of each individual is provided in Supplementary Data 1. The sample represents the Christian Period population living at the site of Kulubnarti between approximately 650-1000 CE. Additional previously-published ancient individuals was used for contextualizing new data. These previously-published data include 263 from Agranat-Tamir et al. 2020, Feldman et al. 2019, Haak et al. 2015, Mathieson et al. 2015, Mathieson et al. 2018, Lipson et al. 2017, Narasimhan et al. 2019, Harney et al. 2018, Lazaridis et al. 2014, Lazaridis et al. 2016, Lazaridis et al. 2017, Olalde et al. 2019, Schuenemann et al. 2017, van den Brink et al. 2017; 967 previously-published modern individuals from Fan et al. 2019, Hollfelder et al. 2017, Mallick et al. 2016, Meyer et al. 2012, Pagani et al. 2012, Pruefer et al. 2013, Skoglund et al. 2015, The 1000 Genomes Project Consortium 2015.
Sampling strategy	We sampled available bones from 111 ancient individuals from Kulubnarti in May 2015. Sampling was carried out by the first author (Kendra Sirak); co-senior author Dennis Van Gerven was also present. Our sampling procedure - in which we selected primarily disarticulated petrous bones from fragmented cranial remains - was aimed at minimizing damage to the collection of remains from Kulubnarti. We extracted DNA from the cochlea (part of the petrous part of the temporal bone) and obtained data that was of sufficient quality for population genetics analysis from 66 individuals. We targeted approximately 1.24 million genome-wide SNPs, which effectively cover almost all independent loci (due to linkage disequilibrium) and provide good power in population history analyses. We combined our new genetic data with previously published genetic data from both ancient and present-day individuals in order to contextualize our data and investigate our research questions at a high resolution.
Data collection	DNA from the ancient remains was extracted, sequenced, and processed into SNP genotype calls. DNA extraction and preparation of sequencing libraries took place in dedicated ancient DNA facilities at Harvard Medical School. These facilities adhere to stringent anti-contamination protocols (including the use of chemical cleaning and sterilization by UV-irradiation, positive air pressure, and accessed only by trained expert technicians wearing full PPE).
Timing and spatial scale	Ancient individuals were sampled from two neighboring cemeteries at the site of Kulubnarti. Ancient individuals lived between 650-1000 years before the present. All samples were collected in May 2015 during the visit of the first author (Sirak) to the collections at the University of Colorado at Boulder maintained by a co-senior author (Van Gerven). DNA extraction and preparation and sequencing of prepared, enriched, and captured libraries took place between 2018-2019.
Data exclusions	45 of the sampled skeletons did not yield working data as assessed by pre-established ancient DNA quality criteria.
Reproducibility	Quality control metrics are reported for the data generated for individual in Supplementary Data 2. To maximize the robustness of our analysis, any individuals with data that did not meet quality control thresholds or a minimum coverage (at least 20,000 SNPs) was excluded from population genetics analysis (thresholds discussed in Methods section).
Randomization	Samples were grouped based on genetic similarity determined through f4-statistics
Blinding	Analyses were performed either for all individuals separately or in groupings by cemetery (with statistically significant genetic outliers excluded); other sample-specific features were not relevant to results. Blinding was not applicable to this study since the metadata associated with each sample was key to exploring relevant research questions.

Did the study involve field work? ☐ Yes ☒ No

Reporting for specific materials, systems and methods

We require information from authors about some types of materials, experimental systems and methods used in many studies. Here, indicate whether each material, system or method listed is relevant to your study. If you are not sure if a list item applies to your research, read the appropriate section before selecting a response.

Materials & experimental systems

n/a	Involved in the study
☒	☐ Antibodies
☒	☐ Eukaryotic cell lines
☐	☒ Palaeontology and archaeology
☒	☐ Animals and other organisms
☒	☐ Human research participants
☒	☐ Clinical data
☒	☐ Dual use research of concern

Methods

n/a	Involved in the study
☒	☐ ChIP-seq
☒	☐ Flow cytometry
☒	☐ MRI-based neuroimaging

Palaeontology and Archaeology

Specimen provenance

The samples that were analyzed as part of this study were excavated in 1979 from the site of Kulubnarti in Sudan; the site is located approximately 120 kilometers south of the present-day Sudanese city of Wadi Halfa. What we consider the site of Kulubnarti includes both the large trapezoidal island of Kulb (dimensions approximately 1 km by 2 km) as well the adjacent west bank. Human skeletal remains were recovered from two cemeteries at Kulubnarti. Excavation of the two contemporaneous and geographically-proximate cemeteries, one on Kulubnarti island and one on the western bank opposite the south end of the island, from which the skeletal remains analyzed in this study derive occurred as part of the United Nations Educational, Scientific, and Cultural Organization (UNESCO) International Campaign to Save the Monuments of Nubia. Site 21-S-46 (the 'S cemetery') was situated within a dry ancient wadi near the west side of Kulubnarti Island, and site 21-R-2 (the 'R cemetery') was located on the mainland's west bank. Detailed information about the site of Kulubnarti and the Kulubnarti cemeteries can be found in Supplementary Note 1.

Excavation of human remains from the two Kulubnarti cemeteries occurred in 1979 under a license granted by the Sudan Antiquities Service (now the National Corporation for Antiquities and Museums) to Dr. William Y. Adams; the excavation of the cemeteries was funded by the National Science Foundation (Grant No. 77-270210-535), and led by Dr. Dennis Van Gerven, co-senior author on this work; it was undertaken as a part of the UNESCO International Campaign to Save the Monuments of Nubia. The excavation was inspected by a representative of the Sudan Antiquities Service monthly, and all excavated remains were reinspected prior to their export, which occurred in accordance with the regulations at that time. A full ethics statement is included in the main manuscript.

Specimen deposition

The skeletal remains studied as part of this work are currently curated at Arizona State University in Tempe, AZ, USA.

Dating methods

We performed radiocarbon (14C) dating and Bayesian chronological modeling at the Center for Applied Isotope Studies (CAIS), University of Georgia (USA) for 29 individuals from Kulubnarti that yielded genome-wide data. See Supplementary Data 2 for results and Supplementary Note 2 for details regarding radiocarbon dating and modeling. Modeled dates in Supplementary Fig. 1.

☒ Tick this box to confirm that the raw and calibrated dates are available in the paper or in Supplementary Information.

Ethics oversight

Excavation of human remains from the two Kulubnarti cemeteries occurred in 1979 under a license granted by the Sudan Antiquities Service (now the National Corporation for Antiquities and Museums) to Dr. William Y. Adams; the excavation of the cemeteries was funded by the National Science Foundation (Grant No. 77-270210-535), and led by Dr. Dennis Van Gerven, co-senior author on this work; it was undertaken as a part of the UNESCO International Campaign to Save the Monuments of Nubia. Prior to the excavation of the Kulubnarti cemeteries, the head of the Sudanese Antiquities Service approved the research plan, including the invasive investigations (such as biochemical analyses) anticipated at the time. The excavation was inspected by a representative of the Sudan Antiquities Service monthly, and all excavated remains were reinspected prior to their export, which occurred in accordance with the regulations at that time.

No further ethical oversight was required as the material was sampled following procedures aimed at minimizing destruction and damage, following discussion with and agreement of those in charge of maintaining the collection.

Note that full information on the approval of the study protocol must also be provided in the manuscript.
